# The Role of Lingual Bone Grafting in the Treatment of Displaced Edentulous Mandibular Fracture with Severe Atrophy 

**Published:** 2018-01

**Authors:** Amin Rahpeyma, Saeedeh khajehahmadi

**Affiliations:** 1 *Oral &Maxillofacial Diseases Research Center, School of Dentistry, Mashhad University of Medical Sciences, Mashhad, Iran.*; 2 *Dental Research Center, School of Dentistry, Mashhad University of Medical Sciences, Mashhad, Iran.*

**Keywords:** Bone graft, Edentulous mandible, Fracture

## Abstract

**Introduction:**

Treatment of edentulous atrophic mandible with severe atrophy is a challenge based on local conditions and systemic diseases confronted by the age group affected. If bone grafting is indicated, it is often used in lateral or inferior aspects of the mandible.

**Case Report:**

A 70-year-old male with a unilateral fracture of the atrophied left mandibular body was managed by lingual bone grafting and inferior border miniplate fixation to prevent two common problems after fracture healing; the need for plate removal before denture fabrication and facial asymmetry in the unilateral body fracture.

**Conclusion:**

If bone grafting is indicated in the management of displaced edentulous mandibular fracture with severe atrophy, a combination of plating at the inferior border of the mandible and lingual corticocancellous bone grafting should be considered in treatment planning.

## Introduction

Edentulous atrophic fractures are not common in the routine practice of maxillofacial surgery. The incidence of this condition is between 1% and 3% of facial fractures (1,2). An atrophic edentulous mandibular fracture is defined as one in which the vertical height of the mandible fracture line is less than 20 mm (3). It is measured perpendicular to the tangent of inferior mandibular border in the fracture site (4). When this height reaches 1 cm or less, complications increase (5). A small contact area in the fracture site, a high proportion of cortical bone, and a reduced healing capacity are negative points in a surgeon’s decision to treat this type of fracture. Bone grafting adds stability to the fracture line, helps fracture union, and helps prevent recurrent fracture.

Two common problems that can occur after fracture healing when buccal bone grafting is used for fracture management are a noticeable facial asymmetry in unilateral fracture treatments and the need for plate removal. When bone grafting is considered in treatment planning, it is often used in the buccal side. The plate is contoured over the graft, such that it presses the graft onto the lateral surface of the mandible. Especially designed preformed titanium plates are also used for this purpose (6).The volume of the bone graft as well as the contoured mini-macro plate that is used for graft fixation in the lateral aspect of the mandible often leads to asymmetry of the face. If the patient wishes to wear removable dentures, it is mandatory to remove the fixation device after fracture healing, under general anesthesia.

## Case Report

A 70-year-old male with a unilateral fracture of the atrophied left mandibular body arising from a car accident was referred to the authors. The patient had controlled diabetes and hypertension. The fracture was displaced unfavorably and was beyond the borders of the complete lower denture seating area. Upon intraoral examination, intact mucosa was seen over the stepped mandible, and upon extraoral examination facial asymmetry was observed.

**Fig 1:**
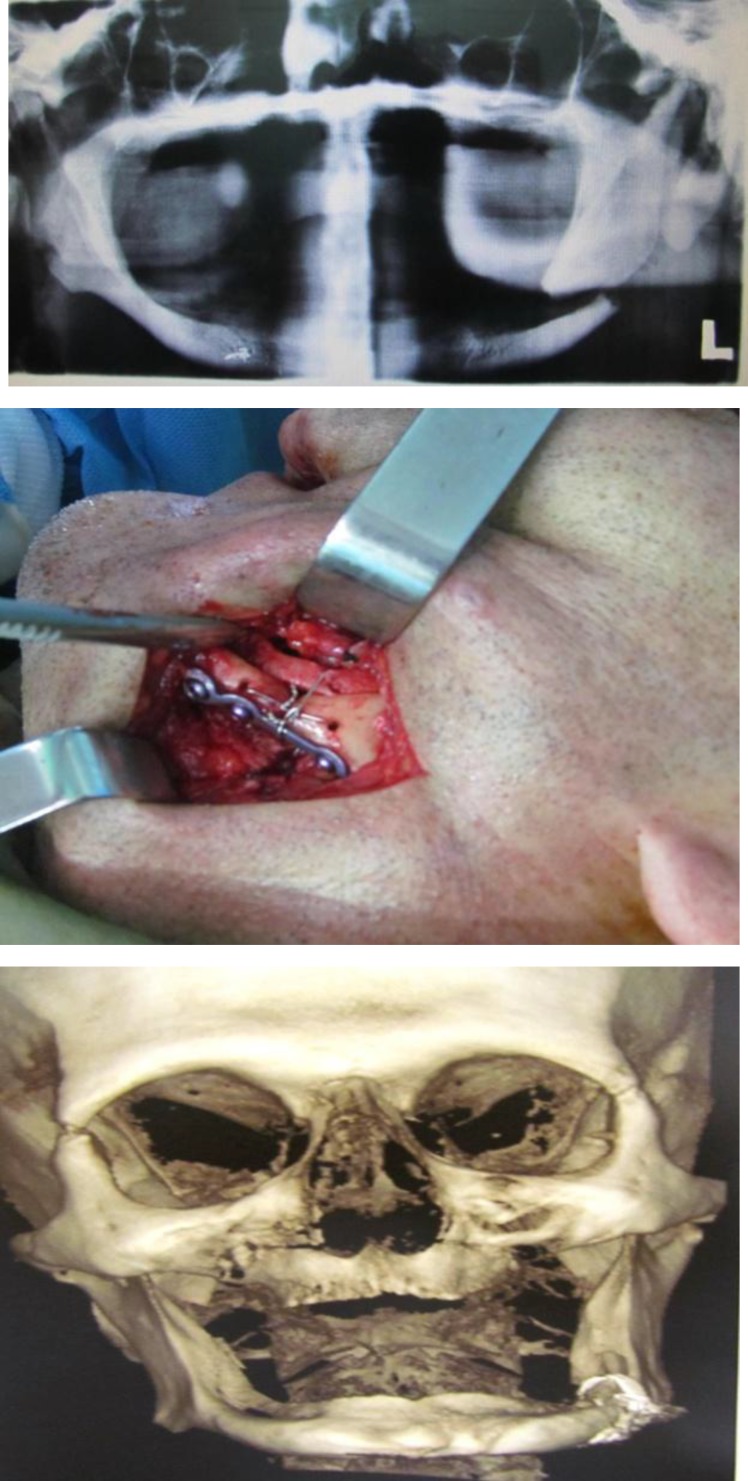
**:** a, Left mandibular body fracture in atrophic edentulous mandible; b, Miniplate at the inferior border of mandible used for internal fixation of fracture. The lingual bone graft is secured to the mandible by transbuccal wire osteosynthesis and circummandibular wiring is used to secure onlay bone graft; c, Post-operative graphy

With an extraoral approach through a submandibular skin incision, the fracture site was approached. After anatomic reduction of the fracture, an inferior miniplate with three screw holes in each side of the fracture was used for internal fixation. A corticocancellous bone block was obtained from the anterior iliac crest and was secured to the lingual cortical plate with the aid of a transbuccal wire. Another autogenous bone block was used for onlay augmentation (Fig. 1).

The post-operative period was uneventful. The surgical wires were removed under local anesthesia 3 months later, and the patient was referred to a prosthodontist for fabrication of new dentures. The patient is under follow up for 3 years.

## Discussion

A bony buttressing effect on the fixation strength of a fractured atrophic edentulous mandible predominantly depends on the remaining height of the mandible (7). A complication rate as high as 20% has been reported after treatment of fractures of the edentulous mandible (8). In severely atrophic cases, free bone grafting has been recommended (9). Buccal bone grafting is a common practice when the augmentation of the mandible is desired locally, and inferior bonder grafting is indicated when reconstruction of the atrophic mandible is demanded (10).

Lingual bone grafting has all the advantages of buccal bone grafting but avoids the drawback of facial bulging after fracture healing. Lingual mucosa and mylohyoid muscle cover the bone graft. Gunning splints or pre-existing patient dentures are not needed in this technique. In a severe atrophic mandible, these devices exert unnecessary pressure on the mucosa, with possible ulceration that impacts on the patient and may endanger the bone graft. Periosteal stripping is necessary when a surgeon decides to use an open reduction and internal fixation technique for the treatment of a fracture. Closed treatment in the management of an edentulous atrophic mandible is a conservative method that is indicated in non-displaced fractures. Aggressive approaches with periosteal stripping, rigid fixation and bone grafting are indicated in displaced edentulous severe atrophic mandibular fractures with the ultimate goal of union and simultaneous augmentation of the mandible in order to prevent mandibular refracture. Today, with the aid of antibiotics and appropriate fixative devices, a compromised blood supply from a carefully reflected mandibular periosteum is not a concern.

## Conclusion

If bone grafting is indicated in the management of a displaced edentulous mandibular fracture with severe atrophy, combination of plating at the inferior border of the mandible and lingual corticocancellous bone grafting should be considered in treatment planning.
